# No improvement in depressive symptoms by vitamin D supplementation: results from a randomised controlled trial

**DOI:** 10.1017/jns.2018.19

**Published:** 2018-11-22

**Authors:** Rolf Jorde, Julia Kubiak

**Affiliations:** 1 Tromsø Endocrine Research Group, Department of Clinical Medicine, UiT The Arctic University of Norway, Tromsø, Norway; 2 Division of Internal Medicine, University Hospital of North Norway, Tromsø, Norway

**Keywords:** Beck Depression Inventory, Depression, Randomised controlled trials, Vitamin D, 25(OH)D, 25-hydroxyvitamin D, BDI-II, Beck Depression Inventory-II, PTH, parathyroid hormone, RCT, randomised controlled trial

## Abstract

In observational studies, vitamin D deficiency is associated with depressive symptoms. However, randomised controlled trials (RCT) with vitamin D supplementation have not been conclusive. In the present study 206 subjects were randomised to vitamin D (100 000 IU (2500 µg) as a bolus dose followed by 20 000 IU (500 µg) per week) and 202 to placebo. The Beck Depression Inventory-II (BDI-II) was filled in at baseline and after 4 months at the end of the study. At baseline the mean age was 51·4 and 52·5 years and mean serum 25-hydroxyvitamin D (25(OH)D) 32·5 and 35·1 nmol/l in the vitamin D and placebo groups, respectively. Among the 408 subjects, 193 had a BDI-II score >4, and forty-five had a score >13. Twenty-three subjects were using anti-depressant or mood-stabilising drugs. At the end of the study, there were no significant differences in Δ BDI-II score (score at the end of the study minus score at baseline), regardless of analysing all subjects, subjects with or without psycopharmaca, or if performing subgroup analyses based on baseline and final serum 25(OH)D levels combined with categories of baseline BDI-II scores >4 or >13. In conclusion, we have not been able to demonstrate any significant effect of vitamin D supplementation on depressive symptoms. However, few of our subjects were clinically depressed. Future RCT should include subjects with more severe vitamin D deficiency as well as more depressed subjects than in our study. In such a setting vitamin D may probably be more relevant as an augmenter of standard antidepressant medication/treatment.

Vitamin D is essential for Ca metabolism and bone health^(^^1^^)^, but may also be important for brain development and function^(^^2^^–^^4^^)^. This is reasonable, since vitamin D metabolites may cross the blood–brain barrier^(^^5^^)^, and the vitamin D receptor as well as the enzymes necessary for the activation of vitamin D to its active form 1,25-dihydroxyvitamin D are present in the central nervous system^(^^6^^,^^7^^)^. Furthermore, vitamin D deficiency has been associated with impaired cognitive function and psychiatric symptoms like depression in several observational studies^(^^8^^,^^9^^)^. However, it has been difficult to demonstrate a causal relationship between vitamin D deficiency and depression.

Since sun-induced production of vitamin D in the skin is the main vitamin D source^(^^1^^)^, the low levels of serum 25-hydroxyvitamin D (25(OH)D), which is used as a marker of vitamin D status^(^^1^^)^, could be the result and not the cause of depression. The only way to settle this question is through properly performed randomised controlled trials (RCT), but the results of those performed so far are not conclusive^(^^10^^–^^15^^)^. One reason for the apparent lack of effect of vitamin D supplementation could be that the subjects included were not vitamin D insufficient (serum 25(OH)D < 50 nmol/l)^(^^16^^)^ and, accordingly, no benefit from additional vitamin D was to be expected. Thus, in the nine RCT that met the inclusion criteria for meta-analysis by Gowda *et al*.^(^^13^^)^ on vitamin D supplementation to reduce depression in adults published in 2015, only two RCT had mean baseline serum 25(OH)D below 50 nmol/l. Furthermore, the mean serum 25(OH)D levels in these two studies were as high as 45 and 47 nmol/l^(^^17^^,^^18^^)^, and the effect of vitamin D deficiency on depressive symptoms was therefore not truly tested.

In Tromsø, Northern Norway, large population-based health surveys are performed at 6- to 8-year intervals^(^^19^^)^. The seventh was conducted in 2015/2016 and included serum 25(OH)D measurements in more than 20 000 subjects. We were therefore able to invite a large number of subjects with low serum 25(OH)D levels in an RCT on vitamin D and depressive symptoms. Our main hypothesis was that supplementation with vitamin D would improve depressive symptoms as evaluated with the Beck Depression Inventory-II (BDI-II). Furthermore, we wanted to perform subgroup analyses in the subjects with low baseline vitamin 25(OH)D levels and with high BDI-II scores since these were the subjects where an effect of vitamin D supplementation most likely would be seen.

## Methods

### Subjects and study design

The main endpoint in the vitamin D intervention study was cardiovascular risk factors, and the design of the study and the main endpoint results have been reported in detail^(^^20^^)^. In short, the subjects were recruited from the Tromsø Study, which is a population-based health survey in the municipality of Tromsø in northern Norway at 69° north^(^^19^^)^. The seventh survey was performed in 2015/2016 and all citizens aged 40 years and above (*n* 32 591) were invited, 21 083 attended, serum 25(OH)D successfully measured in 20 922, and 1489 subjects with serum values <42 nmol/l and with age <80 years invited by mail to participate in the present study. The cut-off of 42 nmol/l was chosen as this was estimated to result in a sufficient number of subjects with vitamin D insufficiency where an effect of vitamin D supplementation could be expected^(^^8^^)^. A total of 698 subjects responded and 639 were screened by telephone for the following exclusion criteria: known granulomatous disease, diabetes, renal stones in the last 5 years, serious diseases making the subject unfit for participation, use of vitamin D supplements >800 IU (20 µg) vitamin D per d, use of solarium on a regular basis, and planned holiday(s) in tropical areas during the study period. Women of childbearing potential without use of acceptable contraception were excluded.

A total of 455subjects passed this initial telephone screening and attended the first visit at the Clinical Research Unit at the University Hospital of North Norway where the informed consent form was signed, clinical examinations performed, medical history taken and blood samples drawn. These examinations did not reveal any contraindication for participation in 422 subjects who then attended the next visit within 2–5 d. At this second (non-fasting) visit, the BDI-II was filled in and the study drugs (Dekristol cholecalciferol capsules (20 000 IU; 500 µg); Mibe GmbH) or identical-looking placebo capsules containing arachis oil (Ayanda GmbH & Co. KG) were dispensed. Five capsules were given as a loading dose followed by one capsule each week.

The randomisation was stratified according to sex, vitamin D status in the Tromsø Study (above/below 25 nmol/l), smoking status (current smoker yes/no) and BMI above/below 27 kg/m^2^. All nurses, doctors, other study personnel and study participants were blinded throughout the study. The subjects were asked not to take any vitamin D supplements (including cod liver oil) during the intervention period.

After 4 months the third and fourth visits were performed, identical to the first and the second. Unused medication was returned and counted. Compliance was calculated as the ratio between capsules used (capsules supplied minus capsules returned) and number of weeks between the second and fourth visits. In all, 411 subjects attended the last visit. Among these, 408 subjects (206 given vitamin D and 202 placebo) had complete BDI-II scores both at baseline and at the end of the study and were included in the analyses.

### Measurements

The subjects were asked regarding anti-depressant or mood-stabilising drugs (psychopharmaca), and classified as users/non-users of psychopharmaca. Height and weight were measured wearing light clothing without shoes and BMI calculated as weight (kg) divided by height squared (m^2^). Serum Ca (mmol/l) was analysed by using the Hitachi 917 (Roche Diagnostics), with reagents from Boehringer-Mannheim. Serum parathyroid hormone (PTH) level (pmol/l) was measured using an Immulite 2000 Intact PTH analyser (Siemens Healthcare Diagnostics). Serum 25(OH)D (nmol/l) was measured with an in-house liquid chromatography–tandem MS method that detects both 25(OH)D_3_ and 25(OH)D_2_ and the sum of these is presented as 25(OH)D in the results. The assay has a between-day CV of <9 %, and a within-day CV of < 2 %^(^^21^^)^.

The BDI-II questionnaire consists of twenty-one items, each with four statements^(^^22^^)^. The subjects are asked to choose the statement that best describes their condition during the last 2 weeks. The statements are rated from 0 (normal or least depressed mood) to 3 (most depressed mood). The BDI-II score was obtained by adding all the values together. A score 0–13 is considered as none or minimal depression, 14–19 as mild depression, 20–28 as moderate depression and 29–63 as severe depression^(^^23^^)^. The BDI-II score varies considerably between populations, with mean scores of about 9 and medians of 6–7 in most non-clinical samples^(^^24^^,^^25^^)^.

### Statistical analyses

Normal distribution was evaluated with skewness and kurtosis and visual inspection of histograms and found normal for all parameters except the BDI-II scores at baseline and at the end of the study. However, the Δ BDI-II scores (value at the end of the study minus value at baseline) were normally distributed.

Correlations were evaluated at baseline with Spearman's rho. Comparisons between groups (men/women, smokers/non-smokers, users/non-users of psychopharmaca, and the vitamin D/placebo groups) at baseline were performed with Student's *t* test, *χ*^2^ test or the Mann–Whitney *U* test.

Comparisons between baseline and end of study values within the vitamin D and placebo groups were performed with Student's *t* test or the Mann–Whitney *U* test. Comparisons between the vitamin D and placebo groups at the end of the study were performed with a general linear model with value at the end of the study as the dependent variable, sex and randomisation status as fixed factors, and age and baseline value as covariates^(^^26^^)^. For the BDI-II score the Δ values (which were normally distributed) were used as dependent variables in this analysis. The distribution of the BDI-II scores at the end of the study across categories of end of study serum 25(OH)D (<25, 25–49, 50–74, and >74 nmol/l) was evaluated with the Kruskal–Wallis test.

*P* < 0·05 (two-tailed) was considered statically significant. Data are presented as means and standard deviations or as medians and ranges. All statistical analyses were performed using IBM SPSS version 22 software.

### Power calculation

For the main endpoint of the study, cardiovascular risk factors (systolic blood pressure, serum LDL-cholesterol, insulin resistance (by homeostatic model assessment; HOMA)), a total number of 450 subjects was needed if wanting a power of 0·8 and *P* < 0·05^(^^20^^)^. For depression, the BDI scores were not normally distributed, and a formal power calculation was therefore not performed. We have in a previous vitamin D intervention study in 441 subjects with mean serum 25(OH)D of 52·5 nmol/l found a slight but significant improvement after vitamin D supplementation as compared with placebo^(^^27^^)^. In the present study we aimed to include a similar number of subjects but with substantially lower serum 25(OH)D levels. Assuming that an effect of vitamin D supplementation would be more pronounced in vitamin D-insufficient subjects, 450 subjects were considered to give the study reasonable power. However, we have now performed a *post hoc* calculation using the normally distributed Δ BDI-II values. Assuming a standard deviation for the Δ BDI-II score of 4·3, a power of 0·8 and *P* < 0·05, 408 included subjects should be sufficient to detect a difference in Δ BDI-II of 1·2 between the two groups.

### Ethics

The study was approved by the Regional Committee for Medical Research Ethics (REK NORD 2013/1464) and by the Norwegian Medicines Agency (2013-003514-40). The study is registered at ClinicalTrials.gov (NCT02750293). All subjects gave their written informed consent.

## Results

**Fig. 1.**
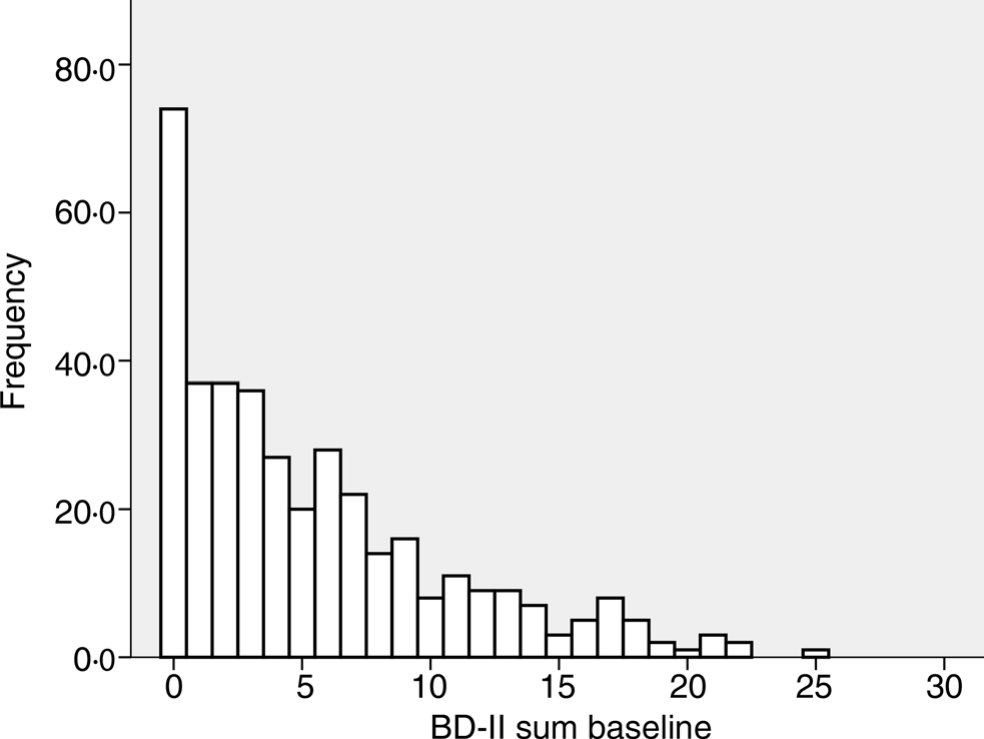
Frequency of Beck Depression Inventory-II (BDI-II) scores at baseline in the 385 subjects not using anti-depressant or mood-stabilising drugs.

**Fig. 2.**
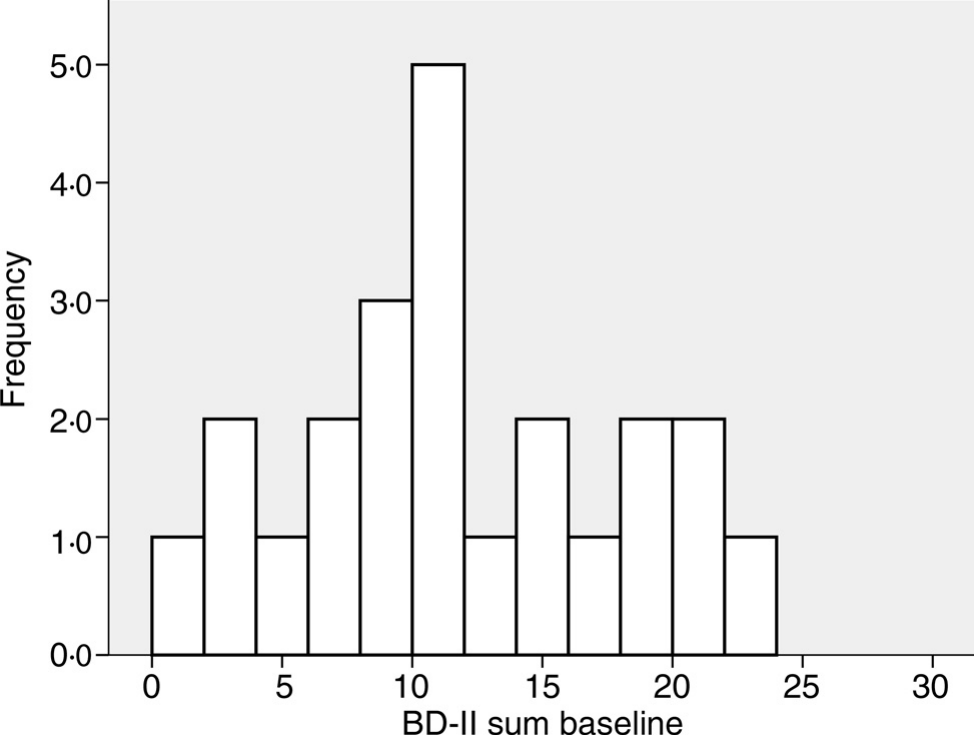
Frequency of Beck Depression Inventory-II (BDI-II) scores at baseline in the twenty-three subjects using anti-depressant or mood-stabilising drugs.

The baseline characteristics of the 408 subjects included in the analyses are shown in Table 1. There were no significant correlations between BDI-II score and age, BMI, serum Ca, PTH or 25(OH)D at baseline, nor were there significant differences between men and women, smokers and non-smokers regarding BDI-II (data not shown). Subjects using psychopharmaca (*n* 23) had significantly lower mean serum 25(OH)D levels than subjects not using psychopharmaca (*n* 385) (28·9 (sd 9·6) nmol/l *v.* 34·1 (sd 12·6) nmol/l; *P* < 0·05), and they also had significantly higher median BDI-II scores (11 (range 1–23) *v.* 4 (range 0–25); *P* < 0·001) (Table 1, Figs 1 and 2).

**Table 1.** Baseline characteristics of all subjects and in those without or with the use of psychopharmaca (Mean values and standard deviations, numbers of subjects, percentages; medians and ranges)

	All subjects (*n* 408)		Subjects not using psychopharmaca (*n* 385)		Subjects using psychopharmaca (*n* 23)	
	Mean	sd	Mean	sd	Mean	sd
Age (years)	52·0	8·8	52·0	8·6	52·0	10·0
Sex (*n*)						
Males	217		205		12	
Females	191		180		11	
Current smokers (%)	22·1		21·0		39·1*	
BMI at baseline (kg/m^2^)	27·8	4·8	27·8	4·8	28·0	4·8
Serum Ca at baseline (mmol/l)	2·27	0·07	2·27	0·07	2·28	0·07
Serum PTH at baseline (pmol/l)	6·7	2·0	6·7	2·0	6·8	2·2
Serum 25(OH)D at baseline (nmol/l)	33·8	12·5	34·1	12·6	28·9†	9·6
BDI-II score at baseline§	4	0–25	4	0–25	11‡	1–23

PTH, parathyroid hormone; 25(OH)D, 25-hydroxyvitamin D; BDI-II, Beck Depression Inventory-II.

* Proportion was significantly different from that of subjects not using psychopharmaca (*P* < 0·05; *χ*^2^ test).

† Mean value was significantly different from that of subjects not using psychopharmaca (*P* < 0·05; Student's *t* test).

‡ Median value was significantly different from that of subjects not using psychopharmaca (*P* < 0·001; Mann–Whitney *U* test).

§ Medians and ranges.

At baseline there were no significant differences between the vitamin D and placebo groups, except for the mean serum 25(OH)D level which was slightly lower in the vitamin D group than the placebo group (32·5 (sd 11·1) nmol/l *v.* 35·1 (sd 13·6) nmol/l; *P* < 0·05) (Table 2). Inclusion in relation to season was also similar in the two groups, with most of the subjects included during the winter months (Supplementary Figs S1 and S2).

**Table 2.** Baseline and end of study values in all subjects and in those without or with the use of psychopharmaca (Mean values and standard deviations; numbers of subjects; medians and ranges)

	All subjects				Subjects not using anti-depressants/psychopharmaca				Subjects using anti-depressants/psychopharmaca			
	Vitamin D group (*n* 206)		Placebo group (*n* 202)		Vitamin D group (*n* 192)		Placebo group (*n* 193)		Vitamin D group (*n* 14)		Placebo group (*n* 9)	
	Mean	sd	Mean	sd	Mean	sd	Mean	sd	Mean	sd	Mean	sd
Age (years)	51·4	8·6	52·5	8·7	51·3	8·4	52·6	8·8	53·0	10·9	50·3	8·8
Sex (*n*)												
Males	109		108		102		103		7		5	
Females	97		94		90		90		7		4	
BMI at baseline (kg/m^2^)	27·8	5·0	27·9	4·7	27·7	5·0	27·9	4·7	29·0	5·3	26·4	3·5
BMI at the end of the study (kg/m^2^)	28·0	5·0	28·0	4·8	27·9	5·0	28·0	4·8	29·3	5·5	26·6	3·4
Serum Ca at baseline (mmol/l)	2·27	0·07	2·27	0·70	2·27	0·07	2·27	0·07	2·28	0·07	2·29	0·09
Serum Ca at the end of the study (mmol/l)	2·29**	0·08	2·27	0·07	2·29**	0·08	2·27	0·07	2·27	0·07	2·25	0·07
Serum PTH at baseline (pmol/l)	6·7	2·2	6·8	1·9	6·6	2·1	6·8	1·9	7·2	2·6	6·2	1·1
Serum PTH at the end of the study (pmol/l)	5·9***	2·0	7·3	2·2	5·9***	1·9	7·3	2·2	6·5	2·2	7·0	1·4
Serum 25(OH)D at baseline (nmol/l)	32·5	11·1	35·1	13·6	32·8	11·2	35·4	13·7	28·3	9·0	29·8	11·0
Serum 25(OH)D at the end of the study (nmol/l)	88·8***	19·5	30·7	9·7	89·3***	18·8	30·8	9·8	82·2***	27·3	28·1	8·0
BDI-II score at baseline†	4·0	0–25	4·0	0–23	4·0	0–25	4·0	0–22	10·5	1–21	14·0	4–23
BDI-II score at the end of the study†	2·0	0–27	2·0	0– 2	2·0	0–27	2·0	0–32	5·5	0–15	9·0	1–27
Change in BDI-II score	−1·5	4·3	−1·9	4·1	−1·4	4·3	−1·9	4·1	−2·8	3·3	−2·6	4·7

PTH, parathyroid hormone; 25(OH)D, 25-hydroxyvitamin D; BDI-II, Beck Depression Inventory-II.

Mean value was significantly different from that of the corresponding placebo group: ** *P* < 0·01, *** *P* < 0·001 (linear regression with baseline value, age and sex as covariates).

† Medians and ranges.

At the end of the study there was an increase in serum 25(OH)D of about 56 nmol/l in the vitamin D group and a decrease in the placebo group of about 4 nmol/l; the serum Ca level was significantly higher and the serum PTH significantly lower in the vitamin D group than the placebo group (Table 2).

**Fig. 3.**
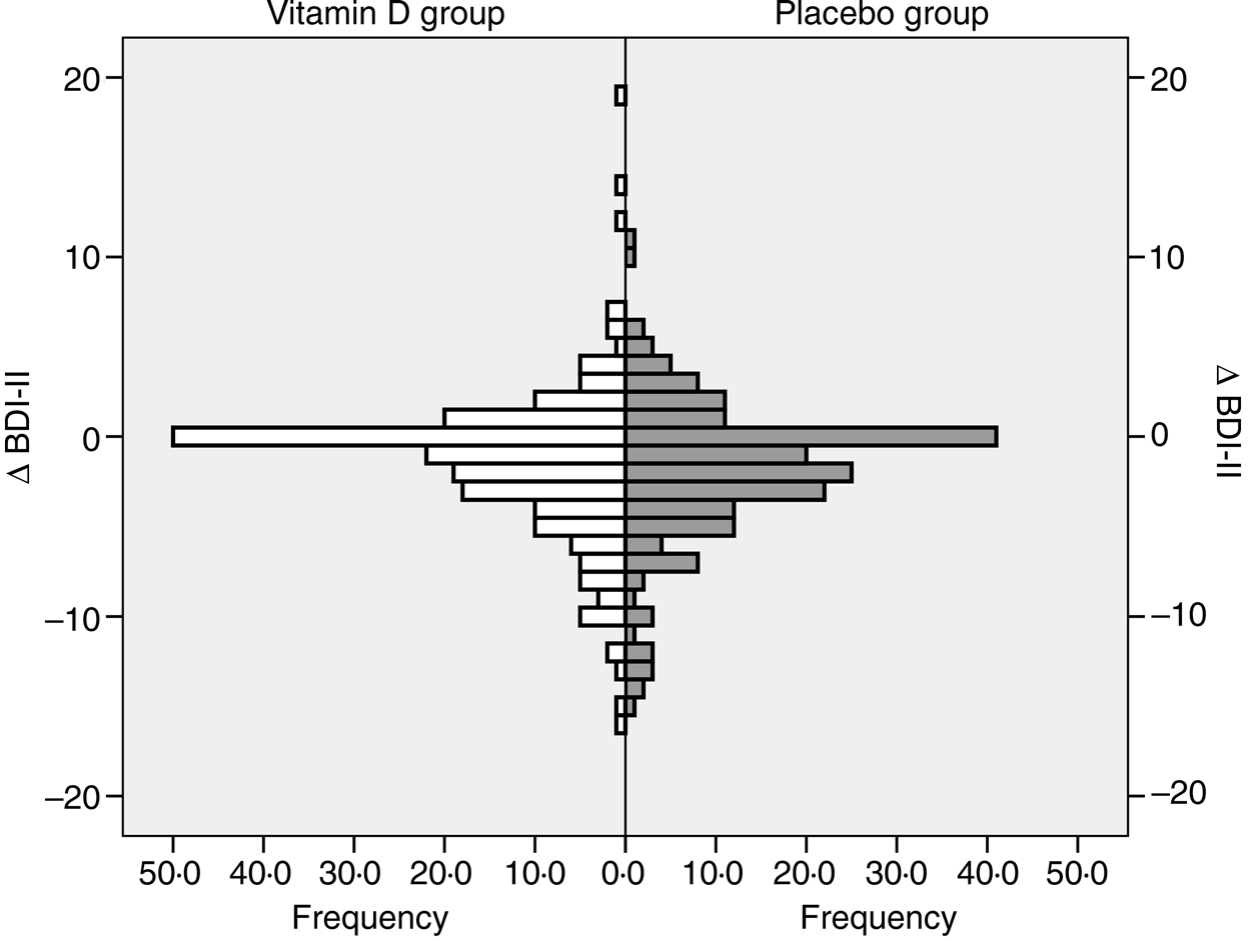
Change in Beck Depression Inventory-II (Δ BDI-II) score (score at the end of the study minus score at baseline) in the vitamin D and placebo groups, all subjects included.

In both the vitamin D and placebo groups the final BDI-II scores were significantly lower than the baseline scores (*P* < 0·05). However, the Δ BDI-II scores did not differ significantly between the vitamin D and placebo group, both when including all subjects and when analysing users and non-users of psychopharmaca separately (Table 2 and Fig. 3).

To test our hypothesis that an effect of vitamin D supplementation would most likely be seen in subjects with low baseline vitamin 25(OH)D levels and with high BDI-II scores, subgroup analyses were performed. Thus, subjects with baseline serum 25(OH)D < 50/40/30 nmol/l combined with final serum 25(OH)D value >70 nmol/l in the vitamin D group, and baseline and final serum 25(OH)D < 50/40/30 nmol/l in the placebo group were analysed separately. However, this did not disclose significant differences in Δ BDI-II regardless of if inclusion was additionally restricted to subjects with BDI-II scores >4 or >13 at baseline (Table 3).

**Table 3.** Change in Beck Depression Inventory-II (Δ BDI-II) scores (score at the end of the study minus score at baseline) in relation to baseline and final serum 25-hydroxyvitamin D (25(OH)D) levels and baseline BDI-II scores* (Mean values and standard deviations)

	Vitamin D group			Placebo group		
		Δ BDI-II score			Δ BDI-II score	
	*n*	Mean	sd	*n*	Mean	sd
All subjects regardless of baseline and final 25(OH)D levels						
Regardless of baseline BDI-II score	208	−1·5	4·3	202	−1·9	4·1
Baseline BDI-II score >4	100	−3·2	5·2	93	−3·8	5·0
Baseline BDI-II score >10	18	−7·1	5·5	27	−4·9	7·4
Subjects with baseline 25(OH)D < 50 nmol/l, and final 25(OH)D >70 nmol/l in the vitamin D group and final 25(OH)D <50 nmol/l in the placebo group						
Regardless of baseline BDI-II score	163	−1·4	4·4	165	−2·0	4·3
Baseline BDI-II score >4	76	−3·1	5·5	76	−4·0	5·2
Baseline BDI-II score >10	15	−6·6	5·5	24	−5·1	7·4
Subjects with baseline 25(OH)D < 40 nmol/l, and final 25(OH)D > 70 nmol/l in the vitamin D group and final 25(OH)D < 40 nmol/l in the placebo group						
Regardless of baseline BDI-II score	136	−1·9	4·2	126	−2·4	4·2
Baseline BDI-II score > 4	66	−3·8	5·0	57	−4·7	4·9
Baseline BDI-II score > 10	14	−7·1	5·4	17	−6·4	6·4
Subjects with baseline 25(OH)D < 30 nmol/l, and final 25(OH)D > 70 nmol/l in the vitamin D group and final 25(OH)D < 30 nmol/l in the placebo group						
Regardless of baseline BDI-II score	74	−1·3	3·6	52	−2·0	4·5
Baseline BDI-II score >4	36	−2·6	4·6	23	−4·4	5·1
Baseline BDI-II score >10	7	−5·4	2·5	7	−6·7	5·4

* Vitamin D analysed *v*. corresponding placebo group with linear regression with baseline value, age and sex as covariates. *P* > 0·05 for all comparisons.

Furthermore, when taking all the 408 subjects together, there were no correlations at the end of the study between Δ BDI-II score and Δ 25(OH)D; nor were there any significant trend for BDI-II score across categories of final serum 25(OH)D values of 0–24 nmol/l (*n* 57), 25–49 nmol/l (*n* 139), 50–74 nmol/l (*n* 52), and >74 nmol/l (*n* 160) (data not shown).

No serious study-related side effects were recorded. Two subjects developed hypercalcaemia (serum Ca = 2·57 mmol/l); one male whose serum Ca normalised upon retesting, and one female who was found to have developed primary hyperparathyroidism. The compliance rate in the study was very high; 14 % of the subjects had a compliance rate between 84 and 100 %, and the rest a compliance rate of 100 %.

## Discussion

In the present study we found both in the vitamin D and placebo groups a significant reduction in depressive symptoms as evaluated by the BDI-II score. However, when comparing the changes in the two groups, no significant effect of the vitamin D supplementation on the BDI-II score was found, even if restricting the analyses to subjects with low serum 25(OH)D levels and mild depression. The reduction in the BDI-II scores from baseline in both groups therefore probably reflects an effect of repeated testing^(^^28^^)^.

We have in two previous studies showed slight, but significant, effects of vitamin D supplementation on depressive symptoms. Thus, in a study on 441 overweight subjects with a mean baseline serum 25(OH)D level of 53 nmol/l, we found a relationship between baseline serum 25(OH)D and BDI score, and a positive effect on BDI in those given vitamin D over a 1-year period as compared with placebo^(^^27^^)^. In the other study, that included 243 subjects with a mean baseline serum 25(OH)D level of 47 nmol/l, the effect was only seen in *post hoc* analyses on those with high BDI score at baseline, and in only one out of the four depression tests employed^(^^18^^)^. In view of our present finding, we do consider our previous reports of positive vitamin D effects on depression as due to chance.

Several meta-analyses and reviews on vitamin D and depression have been published, and there is a general agreement that in observational studies there is an association between vitamin D deficiency and depressive symptoms^(^^10^^–^^15^^)^. However, for intervention studies, there appears to be no clear-cut effect, and many of these studies have been of low quality. Thus, in a systematic review by Spedding^(^^12^^)^ it was reported that out of fifteen RCT included, eight had obvious biological flaws and were not properly designed. This was confirmed in the latest meta-analysis on the topic that included nine RCT with 4923 participants^(^^13^^)^. In none of these nine trials was the mean baseline serum 25(OH)D level below 40 nmol/l, and in only one, where the mean baseline serum 25(OH)D level was 58 nmol/l and the randomisation procedure unclear^(^^13^^)^, were the participant recruited on the basis of clinical depression^(^^29^^)^. The conclusion of that meta-analysis was therefore no surprise: no significant reduction in depression after vitamin D supplementation. It was also recommended that future RCT should be performed among individuals who are both depressed and vitamin D deficient^(^^13^^)^.

In our study we aimed at recruiting the subjects with the lowest serum 25(OH)D levels measured in a population-based health survey (the Tromsø Study) that included more than 20 000 subjects. All the 408 subjects included had a serum 25(OH)D value <42 nmol/l in the Tromsø Study, but since the intervention started up to 4 months later for some of the subjects, not all were vitamin D insufficient (serum 25(OH)D < 50 nmol/l) at baseline. However, in the vitamin D group 163 subjects had baseline serum 25(OH)D < 50 nmol/l and with final serum 25(OH)D > 70 nmol/l (as an indication of adequate effect of the supplementation), and 168 subjects in the placebo group had baseline and final serum 25(OH)D < 50 nmol/l. Among these, thirty-nine subjects (of whom eight were using anti-depressant medication) had a baseline BDI-II score >13 and could thus be classified as being mildly or more depressed. Thus, the ideal study group, depressed, vitamin D-insufficient subjects, was therefore rather small. However, no trend in favour of an effect of vitamin D supplementation was seen, nor when restricting the inclusion to subjects with even lower baseline serum 25(OH)D levels.

We have previously published the cross-sectional relationship between serum 25(OH)D and depressive symptoms (evaluated by the Hopkins Symptoms Check List 10) from the sixth survey in the Tromsø Study with more than 12 000 subjects included in the analyses^(^^8^^)^. Although there was a highly significant relationship, the difference in serum 25(OH)D between those who were in the highest depression score quartile *v.* those in the lowest was only about 6 %. Accordingly, even if there is a causal relationship between serum 25(OH)D and depression, it is unlikely that vitamin D is of major importance. It also follows that a large number of subjects need to be included to demonstrate an effect, especially if those included only have minor depressive symptoms.

In subjects with severe depressive symptoms, it might also be unethical to try treatment with vitamin D alone, even if the subjects are vitamin D insufficient. In clinical trials recruiting such patients, vitamin D would appear to be more suited as an add-on treatment^(^^15^^)^.

As discussed above, our study has weaknesses, in particular since most of our subjects were not clinically depressed. We did not perform a formal power calculation before the start of the study. However, a *post hoc* calculation (that as such should be viewed with caution) indicated that we should have had power to detect a difference in Δ BDI-II score of 1·2 between the two groups. Thus, we should have been able to detect any clinically meaningful effect of the supplementation. As we did eleven subgroup analyses the statistical power of these analyses was considerably reduced, and the subgroup analyses should be considered as exploratory only. We used weekly vitamin D supplementation, and daily doses may be more efficient if the serum level of unhydroxylated vitamin D (cholecalciferol) is important^(^^30^^)^. It has been reported that the relationship between vitamin D and depressive symptoms is only seen during the summer months^(^^31^^)^. This may indicate that vitamin D-independent effects of sunlight may be drivers for the association between 25(OH)D and depression, or perhaps that the relationship is dependent upon serum level of vitamin D (cholecalciferol), that presumably is very low during the winter, and not 25(OH)D^(^^30^^)^. Furthermore, many of our subjects were included during the winter months and therefore came to the final visit during early spring. If season is important and depression less pronounced in the spring, that may also have masked effects of the vitamin D supplementation. We also used only one depression score instrument, which may not have picked up more subtle changes. Also, finally, our study only lasted 4 months, and we cannot exclude that to see an effect of vitamin D a longer intervention period is needed. On the other hand, our study does have strength and importance as it demonstrates the futility of searching for vitamin D effects on depression in cohorts where the depression score is low, even in vitamin D insufficiency.

## Supplementary material

For supplementary material accompanying this paper visit https://doi.org/10.1017/jns.2018.19. click here to view supplementary material
