# Supplementary material for: No improvement in depressive symptoms by vitamin D supplementation: results from a randomised controlled trial
Source: J Nutr Sci. 2018 Nov 22;7:e30. doi: 10.1017/jns.2018.19 (PMC6262688; doi:10.1017/jns.2018.19)
Supplement: Supplementary file 1 [file S2048679018000198sup001.zip › S2048679018000198sup001/JNS 1800019 JORDE Legends to Supplementary figs.docx]

**Supplementary Fig. S1.** Month at baseline visit in the vitamin D group (n = 206) (1 = January, 2 = February, etc).

**Supplementary Fig. S2.** Month at baseline visit in the placebo group (n = 202) (1 = January, 2 = February, etc).
