# Supplementary figures and images for: No improvement in depressive symptoms by vitamin D supplementation: results from a randomised controlled trial
Source: J Nutr Sci. 2018 Nov 22;7:e30. doi: 10.1017/jns.2018.19 (PMC6262688; doi:10.1017/jns.2018.19)

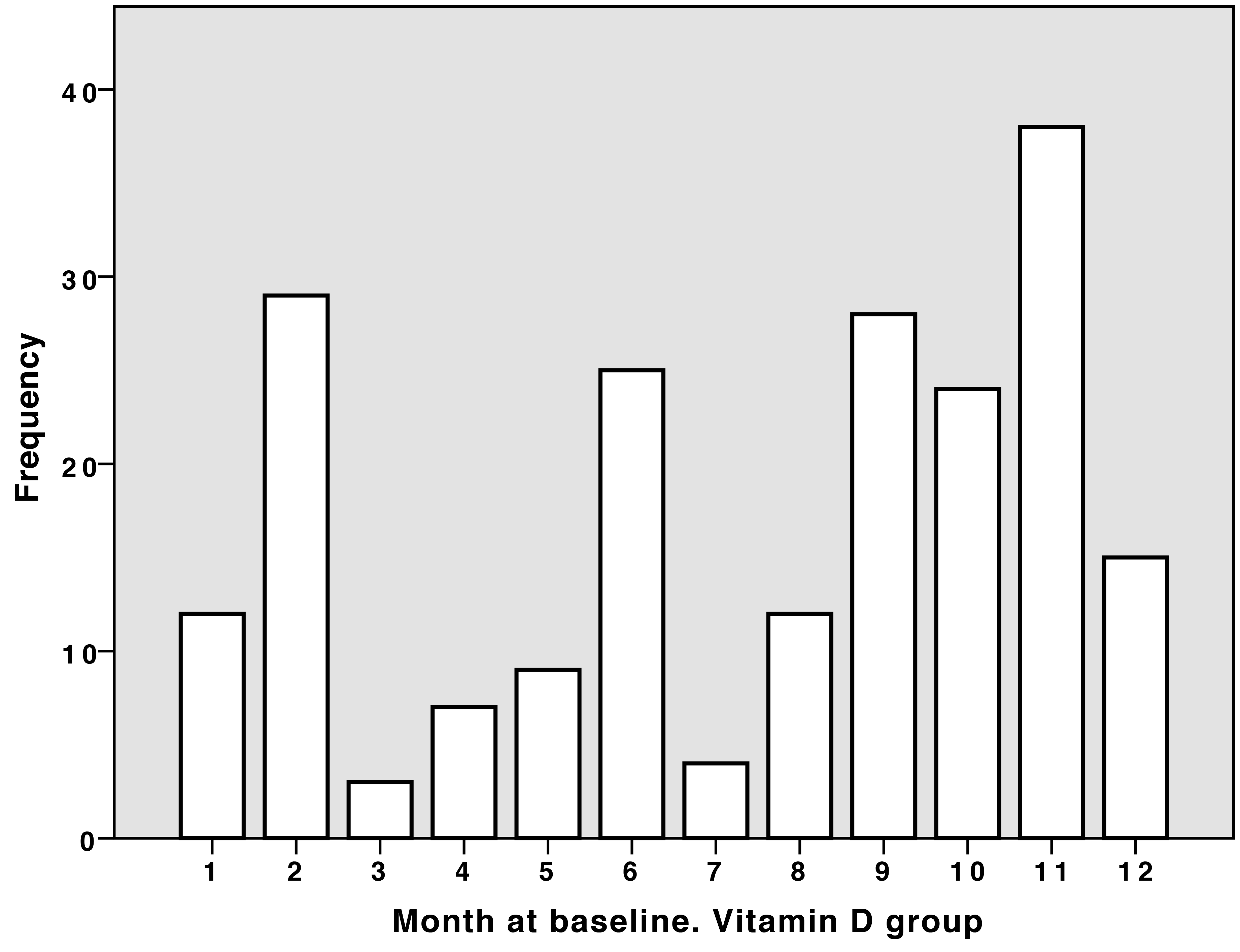

Supplement: Supplementary file 1 [file S2048679018000198sup001.zip › S2048679018000198sup001/JNS 1800019 JORDE Supplementary Fig S1.tif]

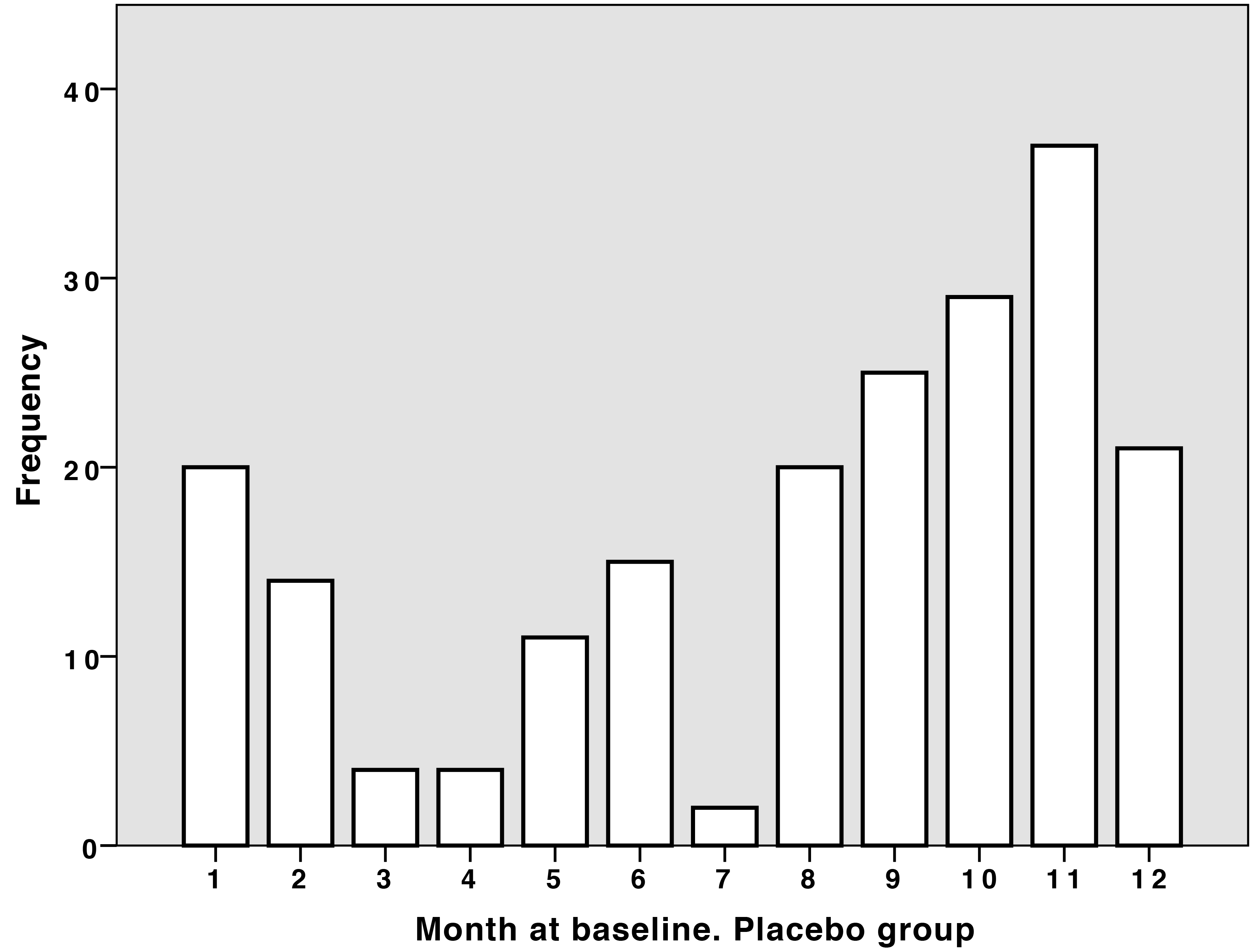

Supplement: Supplementary file 1 [file S2048679018000198sup001.zip › S2048679018000198sup001/JNS 1800019 JORDE Supplementary Fig S2.tif]
